# Quality of Life in Brain Cancer: Clinical Validation of the Mexican-Spanish Version of the EORTC QLQ-BN20 Questionnaire

**DOI:** 10.3389/fneur.2019.00040

**Published:** 2019-01-30

**Authors:** Bernardo Cacho-Díaz, Nydia A. Lorenzana-Mendoza, Luis F. Oñate-Ocaña

**Affiliations:** ^1^Unidad de Neurociencias, Instituto Nacional de Cancerología, Mexico City, Mexico; ^2^Subdirección de Investigación Clínica, Instituto Nacional de Cancerología, Mexico City, Mexico

**Keywords:** health-related quality of life, brain neoplasms, surveys and questionnaires, validation study, prognostic factors

## Abstract

**Background:** Overall survival (OS) of patients with Brain Cancer (BC) is slowly increasing. The disease itself and its treatments deeply impact patient Health-related quality of life (HRQL). Therefore, valid and reliable instruments are needed. In this study, the Mexican-Spanish version of the QLQ-BN20 instrument is psychometrically and clinically validated.

**Methods:** Patients with brain cancer (BC) (primary or metastatic) evaluated at a tertiary cancer center, were invited to respond to the questionnaire, as well as the core-module QLQ-C30. Tests to demonstrate the instrument's internal consistency, the association of HRQL scales with clinical variables and OS were investigated.

**Results:** One hundred and nineteen patients were included in this cohort: 77 women and 42 men (mean age, 46.2 years). Patients answered both instruments in < 30 min.

Good convergent [all correlation coefficients (CC) > 0.37] and discriminant validity was observed and was associated with significant overlap (CC 0.007–0.68). All four multi-item scales of QLQ-BN20 also demonstrated good reliability (Cronbach α > 0.7). Several scales of the QLQ-BN20 were significantly associated with performance status and a modified Recursive Partition Analysis. Of the possible scale correlations, 40 of 161 (24.8%) scales in both instruments, were significantly (directly or inversely) correlated. Visual disorders, Motor dysfunction, Seizures and Weakness of the legs presented association with OS (*p* < 0.05).

**Conclusion:** The Mexican-Spanish version of the BN20 instrument is valid and reliable and can be used in clinical trials in patients with BC. Some HRQL scales were associated with OS and could therefore be incorporated in future studies of prognostic models.

## Introduction

Brain cancer (BC) constitutes a heterogeneous group of diseases, accounting for 1–2% of all primary cancers in adults (1). These neoplasms (primary or secondary) are characterized by severe and complex symptoms, usually associated with a poor prognosis (1, 2). There is no definite cure for most patients. Therefore, a reasonable primary aim of treatment is to extend survival with effective symptom relief (2, 3).

Efforts with emerging new therapeutic strategies are mainly focused on prolonging survival (4). However, BC is associated to symptoms and complications that negatively impact patients' Health-related quality of life (HRQL). BC may directly provoke disabling symptoms including headache, sensory-motor dysfunction, seizures, mood disorders, personality changes, and cognitive dysfunction (5). Consequently, the clinical benefits of treatment should be evaluated not only according to the classical outcome measures (objective response or survival) but also by ensuring HRQL improvement, and must be weighed against treatment side-effects (5–7).

The clinical consequences of disease can be identified by physical examination and evaluated with neurological and neuropsychological tests. Patients' opinions on their own HRQL differ substantially from the opinions of proxies or health-care personnel. Hence, two instruments have been developed to measure HRQL in the specific case of BC: the FACT-Br, Peds-FACT-BrS, and the European Organization for Research and Treatment of Cancer (EORTC) quality of life questionnaire (QLQ) BN20 (8–11). The MDASI-BT is a symptom inventory designed to measure symptoms and not for HRQL assessment (12).

The QLQ-BN20 measures focus more specifically on function and symptoms, while the FACT-Br assessments cover more psychosocial aspects of the disease. Therefore, the QLQ-BN20 is superior when assessing the treatment outcome and may provide more information in trials that focus on functional endpoints, whereas FACT-Br could be more useful in patients with positive functional capacity but psychosocial concerns, although no instrument is superior to the other (13). Both must be considered as complementary.

The QLQ-BN20 instrument comprises 11 symptom scales that cover the more common complains in patients with BC and it was designed to be used with the core questionnaire QLQ-C30. This core instrument comprises five functional scales, and nine symptom scales plus a global HRQL scale. Both have been translated and validated into several languages and have been extensively used in the medical literature. However, available information on the subject published in Latin-American countries is scarce. The aim of this study was to validate the Mexican-Spanish version of the QLQ-C30 and QLQ-BN20 questionnaires in patients with BC.

## Materials and Methods

### Patients

Patients treated at the Neuro-oncology Unit of the *Instituto Nacional de Cancerolog*í*a* (INCan) in Mexico City from February 2005 to October 2014, were invited to participate in this study and respond the questionnaires. Inclusion criteria were: Literate individuals of any gender or age, who had a clinical diagnosis of primary or secondary BC. The diagnosis of BC was established by computed tomography scan and/or magnetic resonance imaging. The clinical history was obtained, as well as blood cytology and chemistry, tumor markers, and chest-X ray. The Karnofsky and ECOG status performance scales were assessed. The Institutional Review Board and Ethics committees approved the study protocol (registration codes 014/007/CCI and CEI 865/14). Patients signed the informed consent form in which the purpose of the study and a safety protection policy were detailed and accompanied the questionnaires.

### Instruments

The EORTC QLQ-C30 consists of 30 items, which are organized into five functional scales (physical, role, emotional, cognitive, and social functioning); three symptom scales (fatigue, nausea and vomiting, and pain); one global health status scale; and six single items (dyspnea, insomnia, appetite loss, constipation, diarrhea, and financial difficulties). The QLQ-BN20 includes 20 items, which are organized into four multi-item scales (future uncertainty, visual disorders, motor dysfunction, and communication deficit) and seven single items (headache, seizures, drowsiness, hair loss, itchy skin, weakness of the legs, and bladder control) (11). The validated Mexican-Spanish version of the QLQ-C30 was used (14). The adapted Mexican-Spanish version of the QLQ-BN20 was pilot-tested in 10 patients with BC to identify the adequacy of the translation. All patients responded the questionnaire without assistance, stated that the questions were clear and easy to understand and complete. Both questionnaires were used with permission of the EORTC Quality of Life Group, and they supervised the entire process.

### Statistical Analysis

Scale scores were calculated by linear transformation of raw scores into a 0–100 score, with 100 representing best global health, best functional status, or worst symptoms, as described by EORTC (15), and the summary score was also calculated (16). Correlation assessment was obtained with the Spearman Correlation coefficient (CC). Convergent validity was determined calculating the CC between each item and items belonging to their own scale, and the success criteria was CC >0.3. Divergent validity was evaluated calculating the CC between each item and items belonging to other scales, and the success criteria was CC < 0.3. Cronbach's α was used to measure multi-item correlation, and the success criteria was ≥0.7. In addition, clinical validity was evaluated by the extent to which scores were able to discriminate among groups of patients who differed in terms of their clinical status. Patients were classified according to treatment intent, the number of metastases (single, multiple or carcinomatosis) and according to Recursive Partitioning Analysis (RPA) (17). Due to the heterogeneity of primary BC and the limited availability of specific prognostic scores for each of the different types of BC, for the purpose of the study, patients with primary BC were classified using the same RPA approach, as if it were metastatic, considering the absence of neoplasm in sites other than the CNS, age (>65 years) and a Karnofsky score >70 (18, 19).

The Kruskal–Wallis test was used to analyze differences between groups. Scale scores were categorized by terciles. The correlation between the different QLQ-C30 and QLQ-BR20 scales was explored to identify differences and clinical overlapping. Overall survival (OS) was considered as the period of time from diagnosis of BC metastases or primary BC to death. The association of HRQL and OS was evaluated using the Kaplan-Meier method, and differences were tested with the Log rank method. Hazard ratios (HR) were calculated using the Cox model. Sample-size calculation was based on the proposal by Tabachnik and Fidell (20); a minimal ratio of 5 patients per item was required (20 × 5), i.e., a sample size of 100 patients. Any probability value of 0.05 or less was considered significant, and two-tailed statistics were applied in all cases. The SPSS for Mac version 23 software was used for computations (IBM, Inc., Armonk, NY, USA).

## Results

### Patients

One hundred twenty-seven (127) patients were invited to participate, but eight did not consent. Therefore, 119 patients with BC were included in the study. There were 77 women (64.7%) and 42 men (35.3%), with a mean age of 46.18 years (SD, 15.8; range 17–80). Brain metastases were found in 76 patients (63.9%); among these, the most common primary cancer sites were: 25 breast (32.9%), 18 lung (23.7%), four ovary (5.3%), four non-Hodgkin lymphoma, three Hodgkin's lymphoma (3.9%), three cervix-uteri, three renal, three testicle, two melanoma (2.6%), two endometrial, one acute myeloid leukemia (1.3%), one gastric, one prostate, one metastases to spinal cord from a treated medulloblastoma, one meningeal metastases from a treated medulloblastoma, one rectum, one nasopharynx, one adenoid cystic and one basocellular skin cancer. Primary BC was found in 43 patients (36.1%) and the most frequent diagnoses were: 12 meningioma (27.9%), six astrocytoma (13.9%), five CNS primary germinal neoplasms, four primary CNS lymphoma, four medulloblastoma (9.3%), four high-grade glioma, three pituitary macroadenoma, two oligoastrocytoma, one craniopharyngioma, one ependymoma, one gliosarcoma, one hemangiopericytoma, one oligodendroglioma, one meningeal sarcoma and one pinealoblastoma. Among patients with primary BC, one developed breast cancer as a second primary, with brain metastases and meningioma; one patient had a retroauricular mucoepidermoid carcinoma with a metachronous astrocytoma and meningioma; in two patients with primary medulloblastoma, one had meningeal metastases and the other developed spinal cord involvement.

Six patients had a 100% KPS, 39 90%, 41 80%, nine 70% and 27 60% or below KPS. All patients answered both questionnaires in < 30 min, and there were 6 missing values (0.1%) in the 50 items of both instruments, including 5,950 possible responses.

### Reliability and Internal Validity

Descriptive statistics of the HRQL data are presented in Table 1. Most scales of both instruments have a zero floor and a 100 ceiling values, whereby mean scores mainly represent high-functional and low-symptom values. The summary of multi-trait scaling analyses is depicted in Table 2; good convergent and discriminant validity is observed for most scales. All multi-item scales presented good own-scale correlations. Divergent validity revealed low correlation for other-scale items but also frequent overlapping. The Cognitive and Pain scales of the QLQ-C30 did not show a Cronbach's α coefficient >0.70, but all four multi-item scales of the QLQ-BN20 did.

**Table 1:** Descriptive statistics of EORTC QLQ-C30 and QLQ-BN20 functional and symptom scales in patients with (primary or secondary) brain cancer (*n* = 119).

	**Mean (SD)**	**Median**	**Floor (%)**	**Ceiling (%)**
**QLQ-C30**				
Global health/QoL	66.96 (28.1)	66.67	0 (1.6)	100 (18.3)
**Functional scales**				
Physical	72.67 (27)	80	0 (1.6)	100 (16.7)
Role	73.61 (32.9)	83.33	0 (8.7)	100 (43.7)
Emotional	72.62 (22.5)	75	0 (0.8)	100 (15.9)
Cognitive	75.56 (19.8)	83.33	16.67 (2.4)	100 (94.4)
Social	73.52 (29.7)	83.33	0 (5.6)	100 (37.3)
**Symptom scales**				
Fatigue	32.24 (23.9)	33.33	0 (11.1)	100 (1.6)
Nausea and vomiting	12.93 (20.9)	0	0 (60.3)	100 (0.8)
Pain	23.11 (24.8)	16.67	0 (34.9)	100 (1.6)
Dyspnea	26.89 (30.5)	0	0 (65.9)	100 (2.4)
Insomnia	26.89 (30.5)	33.33	0 (43.7)	100 (6.3)
Appetite loss	20.73 (29.7)	0	0 (56.3)	100 (5.6)
Constipation	27.45 (30.6)	33.33	0 (43.7)	100 (5.6)
Diarrhea	11.76 (24.8)	0	0 (72.2)	100 (4)
Financial difficulties	47.06 (39.6)	33.33	0 (28.6)	100 (26.2)
Summary score	76.9 (17.04)	81.15	26.11 (0.8)	100 (1.7)
**QLQ-BN20**				
**Symptom scales**				
Future uncertainty	28.36 (25.5)	25	0 (17.5)	100 (1.6)
Visual disorders	24.09 (26.1)	22.22	0 (33.3)	100 (2.4)
Motor dysfunction	26.8 (26)	22.22	0 (27)	100 (0.8)
Communication deficit	21.57 (25.2)	11.11	0 (38.9)	100 (0.8)
Headache	25.58 (29.3)	33.33	0 (43.7)	100 (5.6)
Seizures	5.88 (19.7)	0	0 (84.9)	100 (2.4)
Drowsiness	33.05 (31.4)	33.33	0 (32.5)	100 (9.5)
Hair loss	20.45 (30.41)	0	0 (57.9)	100 (6.3)
Itchy skin	18.77 (27.3)	0	0 (57.9)	100 (3.2)
Weakness of legs	28.01 (30.1)	33.33	0 (40.5)	100 (6.3)
Bladder control	16.25 (27.7)	0	0 (64.3)	100 (4.8)

*n, number of patients; SD, standard deviation; %, is the percentage of patients with floor or ceiling value*.

**Table 2:** Convergent and discriminant validity of scales of the EORTC QLQ-C30 and QLQ-BN20 instruments in patients with (primary or secondary) brain cancer (*n* = 119).

	**Item own-scale correlations^a^**	**Success (%)^b^**	**Item other-scale correlations^a^**	**Success (%)^c^**	**Own-scale multivariate correlations^d^**
**QLQ-C30**					
Global health/QoL	0.787	100	0.06-0.504	51.8	0.888
**Functional scales**					
Physical	0.416–0.658	100	0.053–0.678	47.2	0.875
Role	0.767	100	0.096–0.678	35.7	0.898
Emotional	0.347–0.569	100	0.053–0.389	69.2	0.787
Cognitive	0.19	0	0.02–0.407	66.1	0.289
Social	0.68	100	0.165–0.63	32.1	0.855
**Symptom scales**					
Fatigue	0.348–0.561	100	0.061–0.586	27.2	0.703
Nausea and vomiting	0.671	100	0.024–0.546	66.1	0.795
Pain	0.498	100	0.08–0.525	41.1	0.681
Dyspnea	–	–	0.007–0.46	51.7	–
Insomnia	–	–	0.1–0.399	62.1	–
Appetite loss	–	–	0.202–0.504	6.9	–
Constipation	–	–	0.06–0.407	79.3	–
Diarrhea	–	–	0.007–0.373	96.6	–
Financial difficulties	–	–	0.122–0.468	51.7	–
**QLQ-BN20**					
**Symptom scales**					
Future uncertainty	0.37–0.593	100	0.027–0.633	32.8	0.777
Visual disorders	0.51–0.657	100	0.046–0.496	25.5	0.801
Motor dysfunction	0.556–0.601	100	0.087–0.633	17.6	0.816
Communication deficit	0.609–0.795	100	0.118–0.548	37.3	0.865
Headache	–	–	0.05–0.412	47.4	–
Seizures	–	–	0.046–0.376	94.7	–
Drowsiness	–	–	0.172–0.498	26.3	–
Itchy skin	–	–	0.027–0.502	84.2	–
Hair loss	–	–	0.046–0.502	89.5	–
Weakness of legs	–	–	0.115–0.683	36.8	–
Bladder control	–	–	0.103–0.449	26.3	–

n, number of patients;

a Spearman correlation coefficients;

b *success criteria for item own-scale correlations (>0.3)*;

c *success criteria for item other-scale correlations (< 0.3)*;

d *Cronbach α values. All correlation coefficient values are absolute values*.

### Clinical Validity

Many scales of both instruments were significantly associated with clinically relevant factors. Table 3 describes the association of three categories of ECOG with scale scores of both instruments; nine of Sixteen (including summary score), and four of Eleven scales of the QLQ-C30 and QLQ-BN20, respectively, showed significant associations. Table 4 shows the association of RPA categories (for metastatic and primary BC) with the mean scale scores of both instruments; three of 16 (including summary score), and three of 11 scales of the QLQ-C30 and QLQ-BN20, respectively, did not yield significant associations.

**Table 3:** Mean scale scores of QLQ-C30 and QLQ-BN20 depending on the Eastern Cooperative Oncology Group (ECOG) performance status grading in patients with (primary or secondary) brain cancer (*n* = 119).

	**ECOG 0 (*n* = 14)**	**ECOG 1 (*n* = 67)**	**ECOG ≥2 (*n* = 38)**	***p***
**QLQ-C30**				
Global health/QoL	75 (27.9)	66.8 (28)	63.6 (28.8)	0.333
**Functional scales**				
Physical	91.4 (10.3)	81 (17.8)	51.1 (31.4)	**<0.0001**
Role	95.2 (13.7)	81.7 (24.7)	51.3 (38.4)	**<0.0001**
Emotional	76.2 (16.6)	75.7 (22.1)	65.8 (23.9)	0.089
Cognitive	82.1 (13.8)	78.9 (17.7)	67.1 (22.8)	**0.018**
Social	95.2 (12.1)	78.9 (24.5)	57.9 (34.8)	**<0.0001**
**Symptom scales**				
Fatigue	19.8 (15.8)	29.4 (22.4)	44.7 (24.7)	**0.001**
Nausea and vomiting	5.95 (12.4)	11.5 (19.8)	18 (24.3)	0.223
Pain	17.9 (17.9)	16.4 (20.6)	36.8 (28.5)	**<0.0001**
Dyspnea	2.38 (8.9)	11.9 (20.7)	16.7 (25.4)	0.08
Insomnia	21.4 (24.8)	25.4 (31.3)	31.6 (30.9)	0.43
Appetite loss	9.52 (20.4)	17.4 (26.8)	30.7 (35)	**0.035**
Constipation	26.2 (26.7)	22.4 (28.1)	36.8 (34.5)	0.092
Diarrhea	9.5 (27.5)	7.46 (19.1)	20.2 (30.5)	**0.02**
Financial difficulties	38.1 (34.2)	41.8 (39.5)	59.6 (39.6)	0.065
Summary score	86.7 (10.5)	81.1 (14)	65.9 (18.5)	**<0.0001**
**QLQ-BN20**				
**Symptom scales**				
Future uncertainty	16.1 (12.9)	27.9 (25.3)	33.6 (28.2)	0.179
Visual disorders	5.56 (8.4)	22.4 (24.6)	33.9 (28.8)	**0.001**
Motor dysfunction	10.3 (14.1)	22.6 (25.1)	40.4 (25.2)	**<0.0001**
Communication deficit	22.2 (20)	17.4 (23)	28.7 (29.4)	0.105
Headache	14.3 (17.1)	24.5 (28.7)	31.6 (32.8)	0.238
Seizures	0	3.48 (15.5)	12.3 (27.3)	**0.021**
Drowsiness	21.4 (24.8)	30.8 (31.4)	41.2 (32.4)	0.08
Itchy skin	19 (25.2)	18.4 (29.7)	19.3 (24.1)	0.744
Hair loss	21.4 (28.1)	21.4 (33.2)	18.4 (26.5)	0.866
Weakness of legs	7.14 (14.2)	22.9 (29.7)	44.7 (27.2)	**<0.0001**
Bladder control	7.1 (14.2)	15.9 (27.4)	20.2 (31.5)	0.448

*n, number of patients; numbers represent means (in parentheses are standard deviation values); p, probability values obtained by Kruskal-Wallis test. Bold values correspond to statistical significant values*.

**Table 4:** Mean scale scores of QLQ-C30 and QLQ-BN20 depending on the RPA class in patients with (primary or secondary) brain cancer (*n* = 119).

	**Class I (*n* = 34)**	**Class II (*n* = 58)**	**Class III (*n* = 27)**	***p***
**QLQ-C30**				
Global health/QoL	74.8 (30.1)	64.5 (26.6)	61.4 (27.8)	**0.044**
**Functional scales**				
Physical	89.3 (13.3)	77.5 (19.6)	41.2 (28.4)	**<0.0001**
Role	89.6 (21)	78.7 (25.7)	42.6 (38.8)	**<0.0001**
Emotional	77.5 (19.8)	75.9 (20.9)	59.6 (24.6)	**0.005**
Cognitive	81.1 (17.3)	79 (16.4)	61.1 (23.1)	**0.001**
Social	88.2 (19.9)	77.3 (24.1)	49.4 (36.2)	**<0.0001**
**Symptom scales**				
Fatigue	20.6 (23.6)	31.4 (18.3)	52.7 (23.4)	**<0.0001**
Nausea and vomiting	6.9 (21)	13.3 (18.2)	19.8 (24.5)	**0.01**
Pain	13.7 (19)	19.3 (20.4)	43.2 (29.3)	**<0.0001**
Dyspnea	6.9 (13.7)	12.6 (21.5)	18.5 (28.2)	0.19
Insomnia	18.6 (27.5)	28.2 (29.8)	34.6 (33.9)	0.104
Appetite loss	7.8 (18.5)	20.7 (27.8)	37 (37.4)	**0.001**
Constipation	14.7 (22)	26.4 (27.8)	45.7 (37.2)	**0.002**
Diarrhea	9.8 (25.3)	9.2 (17.4)	19.8 (34.9)	0.45
Financial difficulties	33.3 (37.6)	43.7 (38.1)	71.6 (35.5)	**0.001**
Summary score	86.7 (12.1)	79 (13.4)	60.2 (17.7)	**<0.0001**
**QLQ-BN20**				
**Symptom scales**				
Future uncertainty	21.6 (23.6)	25.7 (22.9)	42.3 (28.8)	**0.009**
Visual disorders	15.6 (20.1)	21.5 (24.2)	40.3 (30.1)	**0.002**
Motor dysfunction	11.1 (18.8)	26.8 (24.2)	46.5 (24.7)	**<0.0001**
Communication deficit	17 (21.6)	18 (22.3)	35 (31.1)	**0.025**
Headache	15 (21.9)	24.1 (27.8)	42 (34.1)	**0.003**
Seizures	2.9 (12.6)	3.4 (14.9)	14.8 (31.1)	**0.05**
Drowsiness	23.5 (31.3)	32.2 (29.3)	46.9 (32.4)	**0.007**
Itchy skin	16.7 (27.5)	19.5 (27.9)	19.8 (26.6)	0.824
Hair loss	16.7 (28.7)	23 (32)	19.8 (29.6)	0.584
Weakness of legs	9.8 (21)	29.3 (28.7)	48.1 (29.7)	**<0.0001**
Bladder control	10.8 (25.6)	16.1 (24.4)	23.5 (35.6)	0.226

*n, number of patients; numbers represent means (in parentheses are standard deviation values); p, probability values obtained by Kruskal-Wallis test. Bold values correspond to statistical significant values*.

### Correlations Between Instruments

Forty-two of 176 (23.8%) possible (16 × 11) bivariate correlations between QLQ-C30 and QLQ-BN20 scales were significant (*p*<0.05). As expected, correlation between functional and symptom scales were usually negative. The correlation matrix is shown in the Table 5.

**Table 5:** Correlation matrix of QLQ-C30 and QLQ-BN20 mean scale scores in patients with (primary or secondary) brain cancer (*n* = 119).

**QLQ-BN20**	**FU**	**VD**	**MD**	**CD**	**HA**	**SZ**	**DW**	**IS**	**HL**	**WL**	**BC**
**QLQ-C30**											
Global health/QoL	−0.474	−0.337	−0.549	−0.365	−0.272	−0.183	−0.46	**−0.074**	**−0.026**	−0.36	−0.214
**FUNCTIONAL SCALES**											
Physical	−0.516	−0.427	−0.69	−0.315	−0.202	−0.24	−0.394	**−0.054**	**−0.07**	−0.591	−0.192
Role	−0.492	−0.424	−0.704	−0.422	−0.187	−0.271	−0.331	**−0.058**	**−0.025**	−0.599	−0.173
Emotional	−0.51	−0.378	−0.373	−0.405	−0.459	**−0.170**	−0.465	−0.249	**−0.141**	−0.293	−0.285
Cognitive	−0.35	−0.366	−0.503	−0.506	−0.353	−0.183	−0.392	−0.211	**−0.039**	−0.354	−0.293
Social	−0.516	−0.377	−0.504	−0.314	−0.308	−0.191	−0.367	**−0.054**	**−0.094**	−0.486	**−0.169**
**SYMPTOM SCALES**											
Fatigue	0.619	0.475	0.661	0.388	0.407	0.248	0.532	**0.166**	**0.076**	0.541	0.257
Nausea and vomiting	0.415	0.387	0.427	0.289	0.223	0.211	0.386	**0.122**	0.225	0.386	**0.148**
Pain	0.319	0.368	0.418	0.29	0.536	0.186	0.385	**0.07**	**0.012**	0.383	**0.149**
Dyspnea	0.454	0.431	0.366	0.352	0.292	0.315	0.412	0.232	0.256	0.241	0.178
Insomnia	0.35	0.183	0.381	0.25	0.278	**0.099**	0.349	**0.162**	**0.15**	**0.179**	**0.123**
Appetite loss	0.484	0.355	0.465	0.299	0.219	**0.174**	0.371	**0.033**	**0.121**	0.436	**0.021**
Constipation	0.272	0.195	0.344	0.209	0.335	0.252	0.287	**0.035**	**0.083**	0.227	0.285
Diarrhea	**0.086**	0.182	**0.117**	**0.114**	0.219	**0.004**	**0.115**	**0.029**	**−0.032**	0.199	0.23
Financial difficulties	0.436	0.337	0.477	0.298	0.217	**0.067**	0.255	**0.084**	**0.104**	0.32	0.217
Summary score	−0.589	−0.58	−0.7	−0.517	−0.424	−0.225	−0.575	**−0.164**	**−0.133**	−0.545	−0.337

*n, number of patients; numbers represent means (in parentheses are standard deviation values); FU, future uncertainty; VD, visual disorders; MD, motor dysfunction; CD, communication deficit; HA, headache; SZ, seizures; DW, drowsiness; IS, itchy skin; HL, hair loss; WL, weakness of legs; BC, bladder control*.

*Non-significant correlations are shown in bold numbers (p > 0.05)*.

### Survival

**Figure 1:**
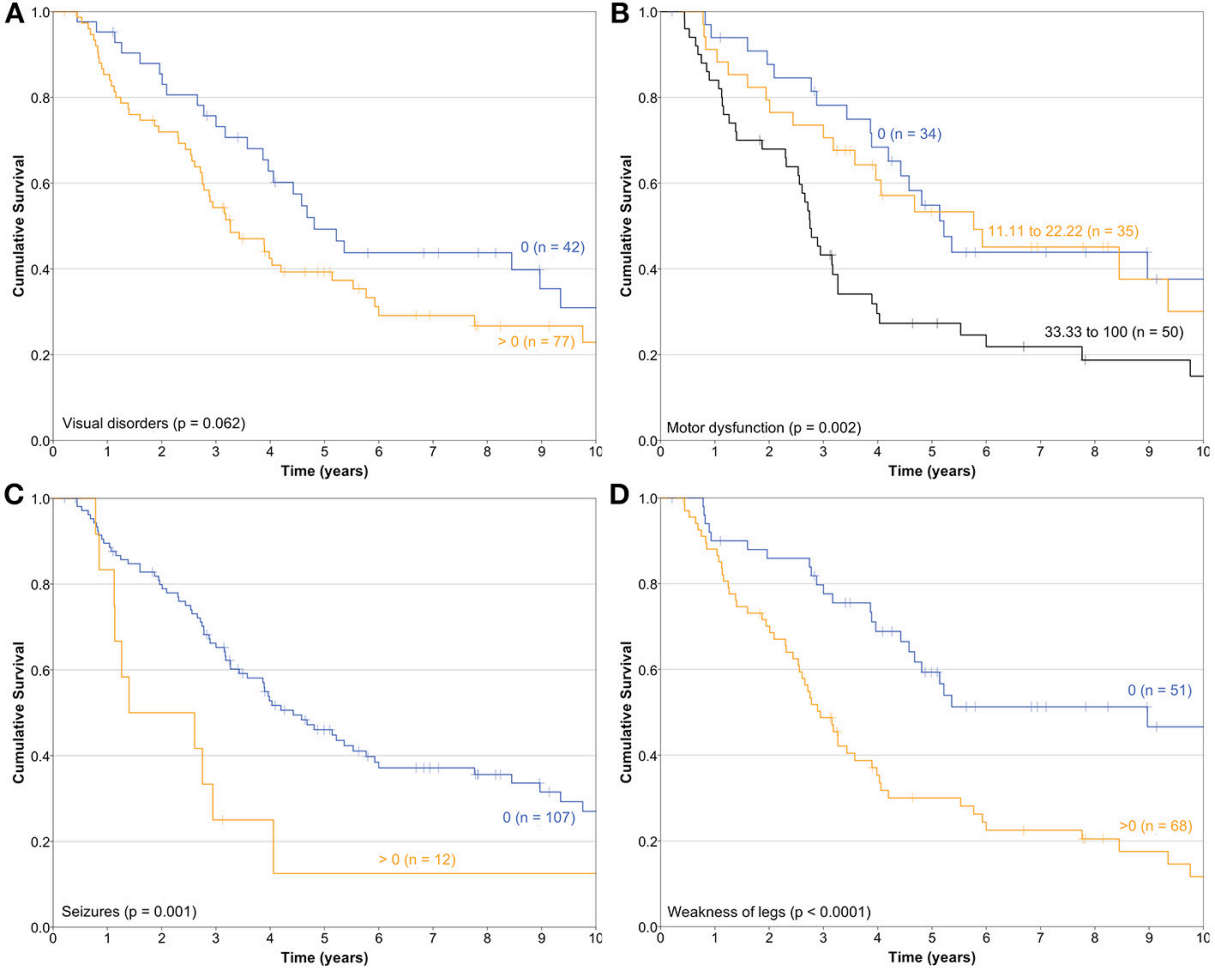
Kaplan-Meier survival curves of the association of health-related quality of life and overall survival constructed depending on **(A)** Visual disorders, **(B)** Motor dysfunction, **(C)** Seizures, and **(D)** Weakness of legs scales of the QLQ-BN20 instrument (*n* = 119).

Median follow-up of the cohort was 4.49 years (SD 3.38) (range 0.21–13.8). During this period, 79 patients (66.4%) died from progressive or recurrent disease. Median OS was 3.98 years (95% CI 2.99–4.97). The bivariate association of HRQL and OS were explored; the physical, role, social, fatigue, nausea/vomiting, pain, dyspnea, appetite loss, financial difficulties scales, and the summary score were associated with OS. Of the QLQ-BN20 scales, Visual disorders (HR 1.01 [95%CI 1.002–1.018]), Motor dysfunction (HR 1.011 [95% CI 1.003–1.019]), Seizures (HR 1.013 [95% CI 1.004–1.023]), and Weakness of the legs (HR 1.013 [95% CI 1.007–1.02]) were associated with OS. The Kaplan-Meier OS curves depending on Visual disorders, Motor dysfunction, Seizures and Weakness of the legs scales are depicted in Figure 1.

## Discussion

In this study, the Mexican-Spanish version of the QLQ-BN20 instrument along with its core instrument QLQ-C30 has been psychometrically and clinically validated as we found them reliable and valid.

The main traditional outcome measures in oncology research are the frequency of objective responses after therapy, progression-free survival, or OS. However, many brain neoplasms are incurable, and maintenance or improvement of patients' HRQL are, at least, as important as increases in the progression-free survival or OS. On the other hand, a patient-centered approach complementing the decision-making process in Neuro-Oncology is feasible and desirable. Most patients with BC can participate actively in the decisions on their management options if relevant information is presented in a clear and reasonable manner. When informed, most patients are able to identify concepts of HRQL, the capability to maintain functional independence and the influence of treatment on survival as the most relevant factors in determining their decision (21). As physicians, we must be prepared to facilitate this process.

In a 20-year period, only five Randomized clinical trials (RCT) included HRQL evaluations as primary or secondary outcome measurements. However, the quality of reporting HRQL data has not considerably improved (22). In these contexts, the availability of valid instruments to accurately measure HRQL is mandatory. In general terms, the psychometric characteristics of our study were similar to the original report and other validation reports (23, 24).

The original QLQ-BN20 instrument was developed in multilingual and multicultural settings in Europe (including European-Spanish) and has proven to be valid and reliable (11, 25). Our study is the first validation protocol of the instrument in the Mexican-Spanish language, performed in a Cancer Center in Latin America. Similar psychometric findings are described in other validation studies but the association of several HRQL scales and relevant clinical variables has not been previously reported (11, 23–26). In known-group comparisons, the association of the QLQ-BN20 instrument with the ECOG performance status scale, RPA and OS are described.

The main pitfalls of our study are that responsiveness to cancer treatment was not investigated as long as we have performed one HRQL evaluation for each patient, and our relatively small sample size (*n* = 119), when compared with the original report from the EORTC (*n* = 891) (11).

Floor and ceiling values are 0–100 in all scales except in the Cognitive scale of the QLQ-C30 (Table 1). In general terms, most patients report high functional and low symptom scales (Table 1), reflecting a population with a recent diagnosis and low disease burden. Convergent and divergent validity is adequate for both instruments, as shown in Table 2, and as similarly reported in the other four validation studies (11, 23–26). The reliability of the Cognitive scale of the QLQ-C30 is below 0.7 and this finding is similar to the Korean validation study (24), while the other three validation studies did not mention the values of the QLQ-C30 scales (11, 23, 25, 26). Low Cronbach α values in terms of Cognitive scale's reliability are frequent in the literature in patients with diverse types of cancer. Examples of this finding include the original EORTC report of the QLQ-C30 instrument (27), and the original Mexican-Spanish validation study of the QLQ-C30 instrument (14). All multi-item scales of the QLQ-BN20 presented fair Cronbach α values as in the other three validation reports (11, 23–26). Certain QLQ-BN20 mean scale scores revealed important associations with ECOG, RPA and OS (Tables 3, 4 and Figure 1). No comparison is possible because to our knowledge, these findings have not been previously reported.

Although, there is currently no available cure for advanced BC, survival rates have been increasing over the last few years, so this tool is useful in assessing the development of an effective treatment that improves HRQL (5). The QLQ-BN20 instrument has been cited in 42 publications in PubMed since 2009 and has been used in clinical trials to measure HRQL in BC patients undergoing chemotherapy (Ch) and/or radiotherapy (RT). A recent study compared two treatment outcomes in glioblastoma patients: one received RT alone, while the other group of patients was treated with RT plus adjuvant temozolomide; QLQ-C30 and QLQ-BN20 were used to assess the patients at follow-up and results showed similar HRQL in the two groups with minimal differences in the nausea/vomiting and constipation scales, which were worse in the Ch / RT group than in the RT only group. Nevertheless, the use of adjuvant temozolomide therapy further prolonged patient survival compared to the RT only group (28).

In clinical trials, statistically significant changes in HRQL can be observed by increasing the sample size or in the scenario of multiple comparisons (such as comparison of multiple HRQL scale scores). However, these changes may not be clinically relevant. The meaning of the minimal clinical important difference is pertinent in the design of clinical trials, when proposing an adequate sample size and in the correct interpretation of results. The minimal clinical important difference can be defined as the smallest difference in the mean score which is clinically important (as in between groups or paired comparison designs). In a recent study, a decrease of 6.1 units or 13.8 units was required to represent clinically relevant deterioration of the Seizures or Weakness of legs variables, respectively (29).

In another study of BC patients, 5.2 units change represented the minimal clinically important deterioration in the motor dysfunction scale. Similarly, 9.1 units change represented clinically important improvement in the communication deficit (30).

In general terms, the authors consider that any 10-unit change or difference in the mean score represents a clinically important difference.

Most patients do not report problems with the cognitive functioning scale. This problem may result from their sociocultural background. At the INCan hospital, we mainly treat patients with a low income, illiteracy, a low education level and poor working possibilities.

Distinguishing patients with glioma from those with meningioma was not tested in this study because of the great variability of histopathology diagnoses in the cohort. This is a validation study, so we did not test the impact of different treatments on HRQL. This question and others could be investigated in future research studies, including the usefulness of these instruments in revealing subtle differences associated to novel treatments in randomized clinical trials.

## Conclusion

In conclusion, the Mexican-Spanish version of the QLQ-BN20 instrument is a valid and reliable test that can be used in clinical studies that include patients with primary or metastatic BC. Some HRQL items were associated with the OS and could be used as prognostic factors or might contribute to assemble prognostic models as aids in treatment trade-offs.

## Author contributions

BC-D and LO-O conception and design of the study and analysis and interpretation of data and draft and revise the article. NL-M recruiting patients, acquisition of data, cleaning of the database. All authors have approved the final version to be submitted.

### Conflict of Interest Statement

The authors declare that the research was conducted in the absence of any commercial or financial relationships that could be construed as a potential conflict of interest.
